# Photosynthetic and Growth Responses of *Arundo donax* L. Plantlets Under Different Oxygen Deficiency Stresses and Reoxygenation

**DOI:** 10.3389/fpls.2019.00408

**Published:** 2019-04-05

**Authors:** Antonio Pompeiano, Thais Huarancca Reyes, Tommaso M. Moles, Lorenzo Guglielminetti, Andrea Scartazza

**Affiliations:** ^1^ International Clinical Research Center, St. Anne’s University Hospital, Brno, Czechia; ^2^ Central European Institute of Technology, Brno University of Technology, Brno, Czechia; ^3^ Department of Agriculture, Food and Environment, University of Pisa, Pisa, Italy; ^4^ Institute of Research on Terrestrial Ecosystems, National Research Council, Pisa, Italy

**Keywords:** anaerobiosis, chlorophyll fluorescence, giant reed, leaf gas exchange, stomatal conductance, mesophyll conductance

## Abstract

Promotion of nonfood species production to marginal, degraded lands abandoned by mainstream agriculture is affected by extremes of water availability (droughts and floods), which have increased in frequency and intensity and account for severe yield reduction. *Arundo donax* L., known as giant cane or giant reed, spontaneously grows in different kinds of environments with limitation to low temperature and is thus widespread in temperate and hot areas around the world. Moreover, this perennial rhizomatous grass has been recognized as a leading candidate crop in the Mediterranean for lignocellulosic feedstock due to its high C_3_ photosynthetic capacity, positive energy balance and low agroecological management demand. In this study, the photosynthetic performance and growth response of *A. donax* to waterlogging and submergence stress following a time course as well as their respective re-oxygenation were analyzed under reproducible and controlled environment conditions. Results of growth response showed that biomass production was strongly conditioned by the availability of oxygen. In fact, only waterlogged plants showed similar growth capacity to those under control conditions, while plants under submergence resulted in a dramatic reduction of this trait. The simultaneous measurements of both gas exchanges and chlorophyll fluorescence highlighted an alteration of both stomatal and non-stomatal photosynthetic behaviors during a short/medium period of oxygen deprivation and re-oxygenation. Photosynthetic CO_2_ uptake was strictly related to a combination of stomatal and mesophyll diffusional constrains, depending on the severity of the treatment and exposure time. Conditions of waterlogging and hypoxia revealed a slight growth plasticity of the species in response to prolonged stress conditions, followed by a fast recovery upon reoxygenation. Moreover, the rapid restoration of physiological functions after O_2_ deprivation testifies to the environmental plasticity of this species, although prolonged O_2_ shortage proved detrimental to *A. donax* by hampering growth and photosynthetic CO_2_ uptake.

## Introduction

The use of marginal lands has gained attention as a sustainable strategy for bioenergy decreasing not only conflicts within food and fuel, but also negative environmental impacts due to indirect land-use change (Gopalakrishnan et al., 2011). To accomplish this goal, it is essential to develop and adopt germplasms that are better able to tolerate abiotic threats, selecting non-food species based on their performance under less than favorable conditions. Perennial rhizomatous grasses are the best candidates as lignocellulosic energy crops because of their high biomass yield and quality, their broad adaptation and tolerance to adverse environmental conditions (Lewandowski et al., 2003).

Hypoxia has recently shown to be a relevant environmental component, thus globally impacting on plant biodiversity and crop production (Pucciariello and Perata, 2017). Events such as strong and frequent precipitation, poor soil quality, slow drainage after over-irrigation, or winter ice encasement limit the oxygen (O_2_) in plants. Such adverse conditions can be intensified by environmental issues, such as flooding, which have dramatically increased in terms of severity and frequency over the past decades (Voesenek and Bailey-Serres, 2015). Limitation of O_2_ occurs normally in plant developmental processes, especially in densely packed and metabolically active tissues such as meristems, seeds, fruits, tubers, and stems (Licausi and Perata, 2009). However, prolonged low O_2_ conditions are harmful for most terrestrial plants, disturbing their growth and resulting in premature death and consequent reduction in yields. In addition, some grass species grown in waterlogged soils or poorly drained areas are susceptible to pests such as *Pythium* spp., *Colletotrichum graminicola* (Ces.) Wils., or *Gaeumannomyces graminis* var. *graminis*, which can develop perfectly under these environmental conditions (Pompeiano et al., 2017a).

Reduced diffusion of gases in floodwaters (−10^4^ fold approximately) limits the availability of O_2_ for aerobic respiration and carbon dioxide (CO_2_) for photosynthesis (Bailey-Serres et al., 2012), being accomplished with diminished light availability for photosynthesis and functional changes in the photosynthetic machinery. The increase in stomatal closure is one dramatic response in plants grown under low O_2_ conditions (e.g. during prolonged waterlogging) (Ahmed et al., 2002). Prolonged or severe stress negatively affects photosynthesis, leading to the accumulation of excess excitation energy *via* light absorption, and thus altering the redox balance and inducing oxidative damage to the photosynthetic apparatus. Since plant growth depends on the supply of carbohydrate and energy from photosynthesis, post-submergence growth recovery may require an efficient acclimation of the photosynthetic apparatus to increased O_2_ and irradiance in order to reduce photo-oxidative damage (Luo et al., 2009).

*Arundo donax* L., also known as giant reed, is a perennial rhizomatous grass of the subfamily *Arundinoideae*. It is well adapted to broad ecological conditions and is dispersed from the Mediterranean basin to subtropical wetlands. This species is mainly riparian, forming robust monospecific stands. *A. donax* has been recently recognized as a leading candidate crop for lignocellulosic feedstock (for the production of energy, fuels and chemicals) due to its high biomass yield and quality, positive energy balance and low ecological/agronomical requirements for its management (Lewandowski et al., 2003; Angelini et al., 2005). Additionally, the levels of nitrogen and water inputs do not affect its above-ground biomass quality composition when used as lignocellulosic feedstock for bioprocessing into fuels (Pompeiano et al., 2013). The species has been characterized by its efficient C_3_ pathway, with high photosynthetic rates resulted from a high capacity for both maximum Rubisco and ribulose-1,5-bisphosphate limited carboxylation rate under light-saturated conditions (Webster et al., 2016). Its ability to fully reinstate photosynthesis after controlled drought stress was observed upon its rewatering, with a rapid restoration of all the key physiological functions (Pompeiano et al., 2017c). Also, during a short/medium period of salt stress, *A. donax* is able to grow without effects on its photosynthetic apparatus, testifying to the environmental plasticity of this species (Pompeiano et al., 2017b).

Recently, a metabolic analysis of *A. donax* exposed to anoxic and hypoxic conditions was performed in a time-course experiment. The species under low O_2_ stress showed a reduction of its absolute growth and alterations in the derived physiological traits in a time-dependent manner (Pompeiano et al., 2015), confirming its ability to cope under the aforementioned stress conditions. Although the responses of giant reed to anoxic and hypoxic treatments showed a similar energy crisis related to the anaerobic metabolism, they differ in the activity of alcohol dehydrogenase and related genes. Overall, the strategy of giant reed under low O_2_ conditions suggested a mechanism where cellular metabolism and growth are restricted and thus plants are able to avoid the stress and endure deep floods.

Along with the abilities to cope with limited O_2_, CO_2_ and energy availability during hypoxia, the capacity to quickly resume normal physiological and metabolic activities upon reoxygenation is an important trait to evaluate stress tolerance (Gibbs and Greenway, 2003). Therefore, to better define the photosynthetic persistence under limited O_2_ conditions and subsequent capacity of recovery of giant reed, our aim was to characterize the short-term dynamic of the post-submergence recovery of growth and photosynthetic performance in plants subjected to waterlogging and hypoxia.

## Materials and Methods

### Plant Material and Growth Conditions

*Arundo donax* L. micropropagated plants of an Italian natural accession (Pisa, IT) were used in the present study. Healthy 10-week-old plantlets were transplanted into 160-hole seed trays (single cell volume 5 cm^3^), filled with a peat-based mix, and then kept in growth chambers for 8 weeks under controlled conditions (22 ± 1°C, 12-h photoperiod, and 800 μmol m^−2^ s^−1^ of light intensity). Plants were daily watered and fertilized weekly with a half-strength Hoagland’s solution (pH 6.50 ± 0.05, EC 1.1 dS m^−1^). Two different treatments were conducted: (1) Waterlogging or soil flooding treatment was carried out flooding the plants with water 2 cm above soil surface; (2) hypoxic or submergence treatment was performed throughout the experiment time using giant reed plants subjected to complete submergence. All treatments were carried out up to 10 days at 22 ± 1°C, 12-h light photoperiod (light intensity: 800 μmol m^−2^ s^−1^). Comparative growth behavior and physiological characterization were performed in three independent, replicated experiments for each experimental condition. For each treatment, nine plants were removed at each time point (4, 7, and 10 days of treatment, DOT) and transferred to the growth chambers. Recovery after oxygen deprivation was evaluated by monitoring the ability of the treated plants to resume growth after returning to control conditions for 10 days [above-ground fresh weight (FW) and dry matter for each plant], and to recover photosynthetic activity after 72 h of reoxygenation. Control plants were kept in the growth chamber during the time course (22 ± 1°C, 12-h photoperiod, 800 μmol m^−2^ s^−1^).

### Chlorophyll *a* Fluorescence and Leaf Gas Exchange Measurements

Gas exchange and chlorophyll fluorescence were measured simultaneously by means of a LI-6400-40 portable photosynthesis system equipped with an integrated fluorescence chamber head (Li-Cor, Lincoln, NE). Measurements were performed on fully expanded leaves after waterlogging and submergence treatment at each time point (4, 7, and 10 DOT) and after 3, 6, 24, and 72 h of recovery. Six individual plants for each treatment and control were selected. Instantaneous measurements of steady state photosynthetic CO_2_ assimilation rate (*A*), stomatal conductance (*g*_s_), intercellular CO_2_ concentration (*C*_i_), transpiration rate (*E*), and actual photon yield of PSII photochemistry (Φ_PSII_) were recorded at a photosynthetic photon flux density (PPFD) of 800 μmol m^−2^ s^−1^, CO_2_ concentration of 400 μmol mol^−1^, relative humidity of about 45–55% and leaf temperature of 22°C. Measurements were taken at steady-state when gas exchange and fluorescence parameters were stable (about 3–5 min). The values of Φ_PSII_ in the light were determined as $\Phi_{\mathrm{PSII}}=\left(F_{m}^{\prime }–F^{\prime }\right)/F_{m}^{\prime }$ at steady-state, where $F_{m}^{\prime }$ is the maximum fluorescence yield with all PSII reaction centers in the reduced state obtained by superimposing a saturating light flash during exposition to actinic light, and *F*′ is the fluorescence at the actual state of PSII reaction centers during actinic illumination. The actual reduction state of PSII reaction centers, which gives an estimate of the excitation pressure on PSII, was calculated as $1-q_{p}=\left(F_{t}–F_{0}^{\prime }\right)/\left(F_{m}^{\prime }–F_{0}^{\prime }\right)\text{,}$ where *q*_p_ is the photochemical quenching, *F*_t_ the transient fluorescence and $F_{0}^{\prime }$ the minimal fluorescence, with all reaction centers open in the presence of quenching. The potential efficiency of PSII photochemistry was calculated on dark-adapted leaves as described in Fiorini et al. (2016) as *F*_v_/*F*_m_ = (*F*_m_ − *F*_o_)/*F*_m_, where *F*_v_, *F*_o_, and *F*_m_ are the variable fluorescence in the dark, the minimum fluorescence yield in the dark and the maximum fluorescence yield in the dark after application of a saturation flash, respectively. The non-photochemical quenching (NPQ) was determined according to the Stern-Volmer equation as $NPQ=F_{m}/F_{m}^{\prime }-1\text{.}$

The mesophyll conductance (*g*_m_) was estimated using the variable *J* method (Loreto et al., 1992) based on the comparison of the electron transport rate (*J*_f_) calculated by both gas exchange and fluorescence measurements. The *J*_f_ was estimated by fluorescence measurements multiplying *Φ*_PSII_ by the incident light intensity and then correcting for the actual fraction of absorbed light (𝛼) and the distribution of light between the two photosystems (𝛽), as described in Scartazza et al. (2017). The gas exchange algorithm used in the variable *J* method is dependent on the CO_2_ compensation point between photosynthesis and photorespiration (Γ*) and respiration in the light (*R*_l_). Rubisco specific factor estimated for annual herbs was used to calculate Γ* as described by Galmés et al. (2005), while dark respiration, which was taken as a proxy for *R*_l_ (Centritto et al., 2009), was measured on leaves maintained in darkness for at least 10 min. The value of total conductance to CO_2_ (*g*_tot_) was calculated as *g*_tot_ = (*g*_s_ × *g*_m_)/(*g*_s_ + *g*_m_).

### Statistical Analysis

After performing the Shapiro-Wilk test for normality assumption diagnostics, linear mixed-effects models were used to control the effects of experimental runs and blocks (i.e. random variables) while testing the effects of treatment, exposure and recovery time, as well as their interactions, on all response variables. To this end, the lmer function implemented in the *lme4* R package (Bates et al., 2015) was used. The package *lmerTest* was used to estimate the *p* for each of the factors in the model, which apply the Satterthwaite approximation for the denominator degrees of freedom or the *F*-statistic (Kuznetsova et al., 2017). Statistically different means in the other response variables were identified by Tukey’s HSD using the *multcomp* package (Hothorn et al., 2008), and probability levels lower than 0.05 were considered as significant.

To identify relationships among the experimental conditions based on data obtained from post-hypoxia chlorophyll *a* fluorescence and leaf gas exchange data, multiple factorial analysis (MFA) was used, implemented in the R package *FactoMineR* (Lê et al., 2008). MFA was performed in two steps. Firstly, a principal component analysis (PCA) was computed on each data set, which was then “normalized” by dividing all its elements by the square root of the first eigenvalue obtained from of its PCA. Then, the normalized data sets were merged to form a single matrix and a global PCA was performed on this matrix. The individual data sets were then projected onto the global analysis to analyze communalities and discrepancies. Each experimental condition had two partial points corresponding to the trait classes (fluorescence and gas exchange). Traits that significantly contributed to MFA dimensions were used to explain differences among genotypes (*α* = 0.05). The length and the direction of the vectors were directly correlated to their significance within each genotype. All computations were performed with R 3.5.1 (R Core Team, 2018), and the R package *ggplot2* (Wickham, 2009) was used for data visualization.

## Results

Analysis of all the biometric and physiological traits revealed a significant (*p* < 0.05) treatment × exposure time × recovery time interaction. Following that, subsequent data were presented for clarity within each exposure time.

### Growth and Biomass Characterization

**Figure 1:**
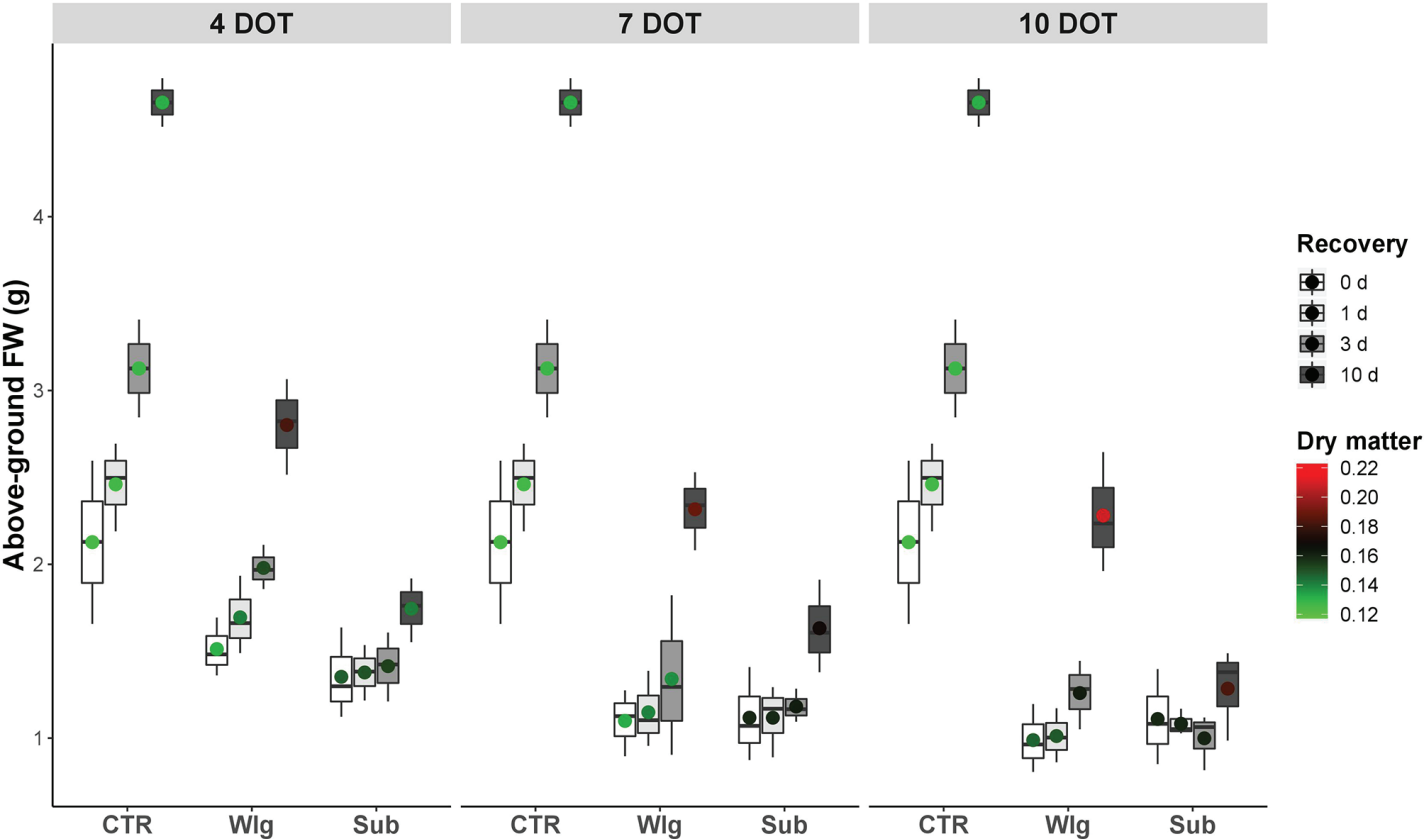
Giant reed (*Arundo donax* L.) biometric traits evaluated as regrowth (above ground FW and dry matter) in a growth chamber up to 10 days at 22 ± 1°C, with a 12-h light photoperiod (light intensity: 800 μmol m^−2^ s^−1^). The dot in the box and whiskers plot indicates the arithmetic mean of the aboveground FW, the color the changes in dry matter.

Under oxygen deficiency, giant reed exhibited increasing susceptibility in terms of above-ground FW as exposure time was prolonged, although the differences were less pronounced for prolonged exposure times, and no significant differences were detected among the two low O_2_ treatments (Figure 1). On the other hand, marked differences in their recovery performance upon reoxygenation were observed compared to normoxic control. Although waterlogging affected above-ground FW, we observed a comparable dynamic of recovery with control plants regardless of exposure time (i.e. after 10 days of recovery, we recorded a 2.1-fold increase on average over 0 days versus 2.2 observed in the normoxia). Also, at 4 days of treatment (DOT), waterlogged plants exhibited rapid regrowth and gradually increased above-ground FW starting from 1 day of recovery. For longer exposure times, as well as for plants fully submerged, giant reed showed no significant increase in above-ground FW until 10 days of recovery.

Under normoxia, dry matter remained very constant throughout the experiment time at ~13%, whereas significant increases occurred under the other conditions (Figure 1). Under waterlogging, plants subjected to 4 and 7 days of stress gradually increased the epigeal dry matter (from control levels to ~19%), whereas for prolonged exposure time a significant increase was observed immediately after returning to control conditions, reaching 21% at the end of the recovery time. A different pattern was observed under submergence, with a slight increase of dry matter compared to normoxic control plants, following which the dry matter remained constantly high for the remainder of the recovery experiment.

### Chlorophyll *a* Fluorescence

**Figure 2:**
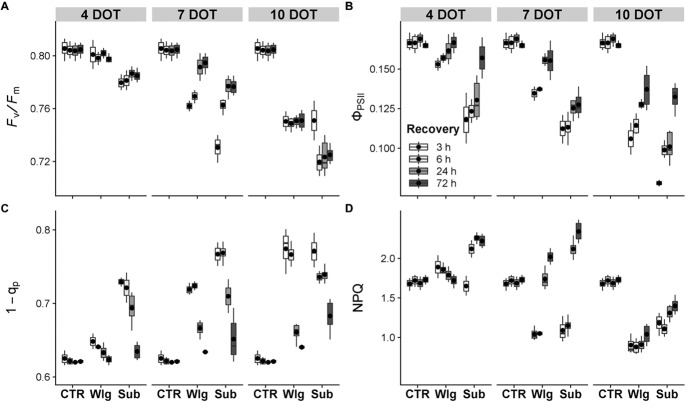
Fluorescence of chlorophyll *a* in giant reed (*Arundo donax* L.) after waterlogging (Wlg) and submergence (Sub) treatment at each time point (4, 7, and 10 DOT) and after 3, 6, 24, and 72 h of recovery for each treatment and control (CTR). **(A)** Potential efficiency of PSII photochemistry (*F*_v_/*F*_m_); **(B)** actual photon yield of PSII photochemistry (Φ_PSII_); **(C)** actual reduction state of PSII reaction centers (1 − *q*_p_); **(D)** non-photochemical quenching (NPQ). The dot in the box and whiskers plot indicates the arithmetic mean of six replicates.

The maximum quantum yield of photosystem II (PSII), as estimated by *F*_v_/*F*_m_ values in dark-acclimated leaves, declined sharply in response to prolonged exposure and more severe scarcity of O_2_ (Figure 2A). Although no significant reduction was observed in 4 days waterlogged plants, a slight decline was detected after prolonged exposure. For instance, at seven DOT, *F*_v_/*F*_m_ significantly declined although a complete and full recovery in *F*_v_/*F*_m_ was visible upon 72 h of reoxygenation. Prolonged exposure caused greater decline, and no recovery was recorded. Submerged plants showed a slight but significant decline starting from four DOT. The species suffered with higher exposure time, although at seven DOT showed a rapid—but not complete—recovery of *F*_v_/*F*_m_ after stress ceased. At the longer exposure level, the plant greatly suffered, especially after 6 h of recovery, and exhibited significantly lower *F*_v_/*F*_m_ levels compared to normoxic controls at the end of the recovery time.

Dynamics of Φ_PSII_ recorded during the recovery showed a similar trend as observed in the *F*_v_/*F*_m_, although a greater sensitivity occurred under stress conditions (Figure 2B). A slight decrease in Φ_PSII_ was visible from the start of waterlogging treatment. At seven DOT marked differences were observed, although the species showed a remarkable ability to restore the PSII photochemistry during recovery. Moreover, a partial recovery was recorded after prolonged oxygen deprivation. Under submergence, the species exhibited an increasing susceptibility to Φ_PSII_ as exposure time was prolonged, and a full recovery was observed only after four DOT. Also, exposure to 10 days submergence treatment exhibited a remarkable ability to partly recovery, which was similar to waterlogged plants.

Observing the dynamics of excitation pressure to PSII, estimated through the 1 − *q*_p_ index, it exhibited increasing susceptibility according to the severity of the treatment and prolonged exposure time (Figure 2C). A complete and full recovery was reached in all plants exposed to prolonged low O_2_ stress, with the exception of the most severe condition. The prolonged exposure until 10 DOT did seemingly affect this parameter, although exposure to submergence treatment resulted in a 10% increase over normoxic levels at the end of the recovery period.

Contrasting variations in NPQ have been observed over time (Figure 2D). At the beginning of the recovery, NPQ showed lower values compared to normoxia under waterlogging and submergence conditions at seven DOT and prolonged exposure times. Moreover, during the recovery we observed a progressive increase in NPQ, reaching higher values compared to normoxia under 7 days of waterlogging and 4–7 days of submergence. For instance, after 10 DOT, NPQ declined by 19 and 40% under submergence and waterlogging, respectively, both presented as the percentage compared to the normoxic control plants after 72 h of recovery. On the other hand, a partial NPQ recovery was detected following both stress treatments after 10 DOT.

### Leaf Gas Exchange Measurements

**Figure 3:**
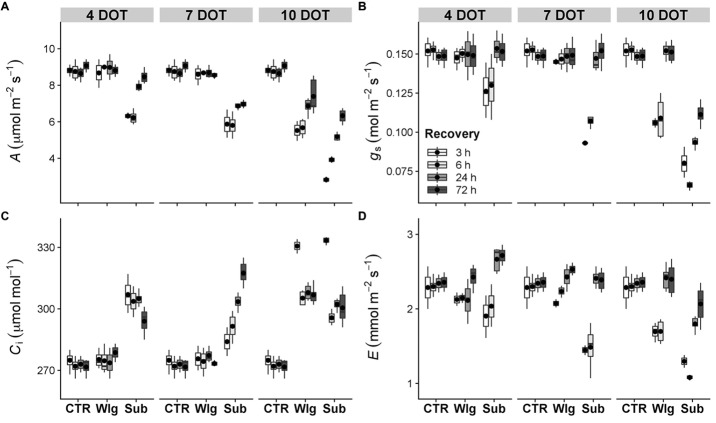
Gas exchanges in giant reed (*Arundo donax* L.) after waterlogging (Wlg) and submergence (Sub) treatment at each time point (4, 7, and 10 DOT) and after 3, 6, 24, and 72 h of recovery for each treatment and control (CTR) were recorded at a photosynthetic photon flux density (PPFD) of 800 μmol m^−2^ s^−1^. **(A)** Photosynthetic CO_2_ assimilation rate (*A*); **(B)** stomatal conductance (*g*_s_); **(C)** intercellular CO_2_ concentration (*C*_i_); **(D)** transpiration rate (*E*). The dot in the box and whiskers plot indicates the arithmetic mean of six replicates.

**Figure 4:**
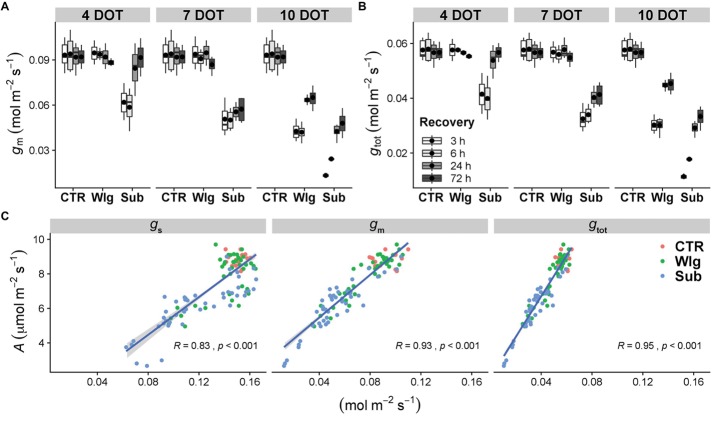
**(A)** Mesophyll (*g*_m_) and **(B)** total (*g*_tot_) conductance to CO_2_ in giant reed (*Arundo donax* L.) after waterlogging (Wlg) and submergence (Sub) treatment at each time point (4, 7, and 10 DOT) and after 3, 6, 24, and 72 h of recovery for each treatment and control (CTR). The dot in the box and whiskers plot indicates the arithmetic mean of six replicates. **(C)** Relationships of CO_2_ assimilation rate (*A*) with stomatal (*g*_s_), mesophyll (*g*_m_), and total (*g*_tot_) conductance. Pearson correlation coefficients (*R*) and significance level (*p*) of the linear regressions are shown.

Changes in leaf gas exchange were recorded at chosen intervals during the time-course experiment. For all the parameters, no significant changes were recorded after waterlogging exposure in the range of 4–7 DOT compared to the control (Figures 3, 4A,B). Furthermore, *A. donax* was able to reach a complete and full recovery after 4 days of submergence treatment for most parameters, with the exception of a partial recovery of *C*_i_ only (Figure 3C). Under waterlogging, a significant reduction in *A* was observed only after 10 DOT, showing a strong but not full recovery in the first 72 h (Figure 3A). Overall, a partial recovery in *A* was recorded in the range of 7–10 days of submergence. Immediately after 3 h of recovery, 10 days-submerged plants exhibited a sharper decline of *A* (−68% compared with the control), whereas observing the dynamics of recovery, it showed a steeper slope in the range of 3–72 h.

Overall, the reduction observed in *A* has been related to a concomitant reduction recorded in *g*_s_ and *E* (Figures 3B,D). Also, we recorded a full recovery in the aforementioned parameters except under the most severe experimental condition, where only partial and significantly lower values compared to control levels were reached at the end of the recovery time.

Significant increases in *C*_i_ were visible from only after 10 days of waterlogging treatment, with more pronounced changes in plants exposed to submergence starting from 4 days of treatment (Figure 3C). After 7 days of submergence, a progressive increase in *C*_i_ was observed in correspondence with an increase of *g*_s_. Under both stress conditions, 10 days of treatments strongly enhanced *C*_i_ followed by a partial recovery.

As expected, *g*_m_ and *g*_tot_ showed an analogous pattern, with significant reductions detected as the stress became more severe (Figures 4A,B). Under waterlogging, significant changes were observed only after 10 DOT, although starting after 4 days under submergence. Marked differences among treatments were observed in kinetic recovery for the aforementioned parameters. A complete recovery was observed for 4 days-submerged plants; meanwhile, prolonged stress conditions caused only a partial recovery. Additionally, under 10 days of waterlogging we recorded only a slight recovery of both parameters after 72 h of re-exposure to O_2_, although reaching higher levels compared with the submerged plants.

Photosynthetic rates of the species were largely determined by conductance to CO_2_ (Figure 4C). Under control conditions, *A. donax* generally exhibited the highest levels of *g*_s_, *g*_m_, and *g*_tot_, while as the stress became more severe the lowest levels were displayed. Assessing the significant relationships between *A* and conductance to CO_2_ from *g*_s_ and *g*_m_ to the combined *g*_tot_ led to an increase in the Pearson correlation coefficient (from 0.83 to 0.95).

### Multiple Factorial Analysis

**Figure 5:**
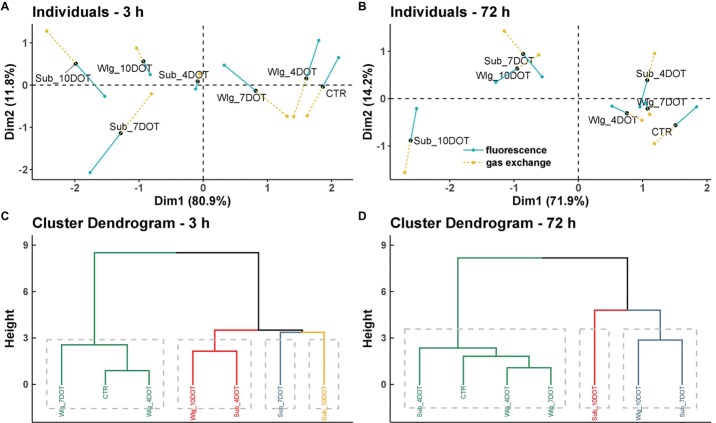
Multiple factor analysis (MFA) of post-anoxia chlorophyll *a* fluorescence and leaf gas exchange data in *Arundo donax* L. plants as affected by incremental oxygen deficiency stress under three exposure times: submergence (Sub), waterlogging (Wlg), and control (CTR). **(A)** Score plot describing the experimental conditions and groups of variables of the two-first principal components recorded at the beginning and **(B)** end of our recovery time. Key: blue “fluorescence”, *F*_v_/*F*_m_, Φ_PSII_, 1 − *q*_p_, and NPQ; yellow gold “gas exchange”, *g*_s_, *g*_m_, *A*, *C*_i_, and *E*. **(C)** Hierarchical clustering of entries based on their traits recorded at the beginning and **(D)** end of recovery time.

MFA revealed the canonical relationship between the experimental condition’s fingerprints (seven entries including two treatments under three exposure times, plus the control) obtained from chlorophyll *a* fluorescence and leaf gas exchange analyses recorded at the beginning (3 h) and end (72 h) of recovery (Figure 5). The coordinates of the two groups of variables were displayed and used to create a map of the groups (data not shown). The coordinates were calculated using the first two dimensions of the MFA (Dim 1 and 2 on the diagram), which resumed 92.7 and 86.1% of the total inertia at 3 and 72 h of recovery, respectively.

The representation of the entries provided by MFA can be read as in a usual PCA (Figures 5A,B; Individuals—3 and 72 h). The coordinates of the descriptors correspond to the correlation coefficients between these variables (chlorophyll *a* fluorescence and leaf gas exchange traits) and the factors (entries). The length and the direction of the vectors are directly correlated to their significance within each experimental condition. The hierarchical clustering provided by each MFA highlighted the overall performance of the entries obtained through the single analysis of the chlorophyll *a* fluorescence and leaf gas exchange data (Figures 5C,D; Cluster dendogram—3 and 72 h). At both recovery times, factorial axis 1 (80.9 and 71.9% of the variance at 3 and 72 h of recovery, respectively) clearly separated the main clusters obtained. At the beginning of the recovery time, the four phylogenetic trees showed that 4 and 7 days-waterlogged plants share more similarity to the control and that 4 days-submerged plants cannot be separated from 10 days-waterlogged plants, while 7 and 10 days-submerged plants are clearly differentiated from the other analyzed treatments (Figure 5C). A different pattern was observed at the end of the recovery time, with the 4 days-submerged plants sharing more similarity to the control group while 10 days-waterlogged plants could not be separated from 7 days submerged plants, and the most extreme treatment (10 days submerged plants) was clearly differentiated from the others on the basis of their chlorophyll *a* fluorescence and leaf gas exchange traits (Figure 5D).

## Discussion

In the present study, biometric alterations and time-dependent alterations in the photosynthetic performance were observed in response to reduced O_2_ availability. As expected, the degree of oxygen deficiency (waterlogging vs. submergence) affected the biometric and physiological response. Overall, our biometric data highlight the ability of *A. donax* to cope with severe hypoxic stress, as well as a rapid recovery upon the cessation of stress under waterlogging conditions, maintaining mostly unaltered regrowth response following treatments lasting up to 10 DOT. Although the present data are not directly comparable to the work performed by Pompeiano et al. (2015) on how the anoxic and hypoxic stress response of the species cause marked differences in ecotype and length of the time-course experiment, previous ecological characterization of the species conducted in riparian habitat confirm the aforementioned perspective (Else, 1996; Bell, 1997).

Reoxygenation stress also triggers a significant drop in hydraulic conductivity in shoots, causing leaf desiccation even in the presence of sufficient soil water (Tamang and Fukao, 2015). Any decrease in hydraulic conductivity in the roots might be due to the regulation of aquaporins in the roots, which occurs when roots are suddenly exposed to hypoxia or anoxia (Setter et al., 2010). Mechanisms regulating shoot dehydration upon recovery also remain to be elucidated. In rice (*Oryza sativa* L.), the flooding tolerance-associated *SUB1A* gene also confers drought and oxidative stress tolerance during reoxygenation through increased ROS scavenging and enhanced abscisic acid (ABA) responsiveness. Following de-submergence, dehydration caused by reduced root function and reoxygenation generates the submergence recovery signals ROS, ABA and ethylene that elicit downstream signaling pathways regulating various aspects of recovery (Yeung et al., 2018).

Chlorophyll fluorescence represents a powerful indicator of stress-induced damage to PSII (Guidi and Calatayud, 2014). A decrease of *F*_v_/*F*_m_ under O_2_ shortage conditions has previously been observed in warm- and cool-season perennial grasses (Pompeiano et al., 2017a). Mauchamp and Méthy (2004) showed that PSII photochemistry in *Phragmites australis* (Cav.) Trin. ex Steudel, a closely related and ecologically similar species to *A. donax*, was affected by submergence and exhibited a different recovery behavior depending on duration and degree of submergence, with completely submerged leaves that did not recover after 1 week. Moreover, the determination of *Φ*_PSII_ has an advantage with respect to *F*_v_/*F*_m_, since it is more sensitive to a large number of stressors including flooding (Ren et al., 2016). The analysis of post-anoxia recovery of the fluorescence indices *F*_v_/*F*_m_ and *Φ*_PSII_ has been previously used for selecting species and cultivars of grasses more able to acclimate their photosynthetic apparatus to oxygen deprivation (Pompeiano et al., 2017a). Overall, these results highlight the sensitivity of the photosynthetic apparatus and PSII photochemical processes to O_2_ deficiency and suggest the use of fluorescence techniques as a fast and reliable tool for studying the photosynthetic responses under waterlogging and submergence conditions and recovery. Accordingly, our results showed that waterlogging and submergence caused a change of both *F*_v_/*F*_m_ and Φ_PSII_ in giant reed during the recovery period, depending on the treatment (waterlogging or submergence) and its duration. In particular, post-submergence and -waterlogging treatments were characterized by a decreased PSII photochemistry followed by a progressive recovery (Luo et al., 2009). The lower Φ_PSII_ in waterlogged and submerged plants during the first hours of recovery can be explained by a rapid enhancement of the reduction state of the PSII primary acceptors (Q_A_ pool), as indicated by the concomitant increased 1 − *q*_p_ values. This parameter is a proxy of the excess excitation pressure at PSII (Scartazza et al., 2016), which needs to be dissipated as heat to avoid photodamage to the photosynthetic apparatus. Hence, the reduced Φ_PSII_ in waterlogged and submerged plants during the first hours of recovery, associated with enhanced 1 − *q*_p_, highlights a decreased efficiency of excitation energy capture by open PSII reaction centers in the light-acclimated state (Roháček and Barták, 1999). It has been suggested that a reduced electron transport capacity during the first hours of recovery after submergence could be beneficial for the plants, preventing irreversible damage due to electron leakage and ROS formation through interaction with oxygen.

Subsequently, an increase of photochemistry activity associated with a decreased reduction state of the PSII reaction centers was observed from 24 to 72 h of recovery, depending on the treatment and duration. Indeed, at the end of the recovery period, both *F*_v_/*F*_m_ and Φ_PSII_ values remained lower than the control starting from 7 to 10 days of submergence and waterlogging, respectively. A significant drop in dark-adapted *F*_v_/*F*_m_ and Φ_PSII_ was previously observed in rice leaves immediately following de-submergence and was attributed to light-mediated inhibition of PSII performance in the submergence-sensitive cultivar (Alpuerto et al., 2016), although these authors observed a full recovery of PSII photochemistry within 24 h after de-submergence.

In order to avoid possible damage to the photosynthetic apparatus, excess light energy must be safely dissipated in thermal energy processes, estimated by means of NPQ. Our data showed a decreased NPQ in the first hours after de-submergence followed by a progressive increase of both Φ_PSII_ and NPQ within 72 h of recovery associated with a reduced 1 − *q*_p_. This suggests a restoration of the ability to dissipate the radiative energy as both photochemical and non-photochemical processes following the waterlogging and submergence treatments. In agreement with these results, Alpuerto et al. (2016) reported a decline of NPQ immediately after de-submergence followed by a rapid recovery in rice and highlighted that a greater capability for NPQ-mediated photoprotection may be crucial for a faster recovery of photosynthetic performance. However, our data indicate that this ability was partly compromised after 10 days of submergence and waterlogging, when both Φ_PSII_ and NPQ showed only a partial recovery and remained lower than control after 72 h of re-oxygenation, possibly leading to excess energy at PSII and, consequently, to photodamage in the reaction centers. This hypothesis is supported by the significant reduction, although slight, of *F*_v_/*F*_m_ in both waterlogged and submergence plants compared to normoxic control, indicating a sustained quenching and, possibly, chronic photoinhibition of PSII. This negative effect on PSII photochemistry was evident starting from 7 days of submergence, when both the maximum and the effective quantum yields of PSII photochemistry were partially impaired after 72 h of recovery. These results, also remarked by the multivariate analysis, suggest that after a relatively long period of submergence or waterlogging (a threshold of 7 DOT for submergence and 10 DOT for waterlogging), plants showed a sustained decrease in photochemistry capacity without a compensatory increase of non-radiative energy dissipation ability, leading to PSII photodamage. This is in agreement with previous works on trees of a tropical seasonally flooded forest (Rengifo et al., 2005) and on off-season flooding *Distylium chinense* (Fr.) Diels (Liu et al., 2014), showing a decreased maximum quantum efficiency of PSII without a compensatory increase in NPQ. Hence, our data suggest that giant reed showed an impairment of the photosynthetic capacity that was due to both stomatal and non-stomatal factors, depending on the treatment (waterlogging or submergence) and its duration.

The appearance of both stomatal and non-stomatal detrimental effects on CO_2_ photosynthetic uptake in *A. donax* was confirmed by the gas exchange analysis. The limited leaf gas exchanges induced a reduction in photosynthetic CO_2_ uptake during the first hours of recovery following submergence (4, 7, and 10 DOT) or waterlogging (10 DOT) treatments, with only a partial recovery to control values after 7 and 10 days of submergence and 10 days of waterlogging. In agreement with our results, Alpuerto et al. (2016) found that the net CO_2_ assimilation rate was reduced by submergence in rice and partially recovered within 24 h after treatment, but it did not recover completely in the more submergence-sensitive rice genotype. The root system may be impaired from waterlogging or submergence, leading to a reduced hydraulic conductance of the roots and inability to take up water from the soil (Shahzad et al., 2016). As a consequence, plants recovering from submergence generally show drought-like symptoms and tend to close stomata in order to reduce water loss through transpiration (Fukao et al., 2011; Alpuerto et al., 2016). Although stomatal closure is the most common response in plants growing under O_2_ deficiency (Ahmed et al., 2002), the reduction of the CO_2_ assimilation rate can also be attributed to non-stomatal factors (Herrera et al., 2008). Our data showed a decrease of *g*_s_ associated with reduced *A* and *E* and increased *C*_i_ during the first hours of recovery. This was evident in submerged plants starting from four DOT, while in waterlogged plants an increase of *C*_i_ with respect to the control was observed after only 10 DOT. According to Farquhar and Sharkey (1982), an increase of *C*_i_ in response to changes in *A* and decrease of *g*_s_ indicates a strong contribution of non-stomatal limitation to carbon photosynthetic uptake. These results are in agreement with Liu et al. (2014), who observed a gradual increase in *C*_i_ with increasing flooding duration in *D. chinense*, and with Yordanova and Popova (2007) who showed that *C*_i_ increased in all the flooded maize plants without significant changes in *g*_s_. The *C*_i_ trend during the recovery period in giant reed was dependent on the coordinated variations of *A* and *g*_s_. For example, after 7 days of submergence, plants showed a full recovery of *g*_s_ from 3 to 72 h of re-oxygenation associated with only a partial recovery of *A*, leading to a progressive increase of *C*_i_ after de-submergence. Non-stomatal constrains to photosynthetic CO_2_ uptake can be due to ROS formation following the over-reduction of PSII reaction centers. Indeed, it has been shown that plants growing under waterlogging or submergence conditions face oxidative damage due to ROS production, which alters membrane integrity and induces damage to the photosynthetic apparatus (Blokhina et al., 2003). Our data showed that negative effects on the photosynthetic apparatus were more pronounced in submerged plants after only four DOT than in waterlogged ones. It has been suggested that injuries in plant tissues and organs developed underwater can be amplified upon de-submergence, because of the sudden increase in O_2_ and light intensity that could exacerbate ROS production (Blokhina et al., 2003). Accordingly, Fernández (2006), studying the fluorescence responses to flooding in leaves of *Pouteria orinocoensis* (Aubr.) Penn. Ined., showed that submerged leaves exhibited chronic photoinhibition, whereas the fluorescence analysis on emerged leaves revealed the occurrence of dynamic, rather than chronic, photoinhibition.

The reduced *g*_s_ in waterlogged plants has been associated with an enhanced leaf ABA content (Jackson and Hall, 1987), although stomatal behavior may also be affected by the impairment of root hydraulic conductivity and permeability due to low O_2_ levels (Else et al., 2001, 2009). Rodríguez-Gamir et al. (2011) observed that ABA concentration in leaves only started to increase after 3 weeks of flooding in citrus seedlings, suggesting that stomatal closure occurs in the absence of a rise in leaf ABA content. The modulation of stomatal closure was attributed to downregulation of the expression of PIP aquaporins. Other than stomatal closure, it has been proposed that plants growing in waterlogged soil may reduce the CO_2_ transfer from the substomatal cavities to the carboxylation sites within the chloroplasts (i.e. the mesophyll conductance to CO_2_ or *g*_m_), leading to a reduction in photosynthetic CO_2_ uptake (Gaur and Sharma, 2013). A negative effect of flooding on *g*_m_ was observed by Moldau (1973) in *Phaseolus vulgaris* (L.). Moreover, Black et al. (2005) showed an alteration of both *g*_s_ and *g*_m_ in waterlogged seedlings of *Picea sitchensis* [Bong. (Carr.)] grown under exposed and shaded conditions. It has been reported that photosynthetic CO_2_ uptake is dependent on a tight mutual regulation between stomatal and mesophyll conductance (Chaves et al., 2002; Scartazza et al., 2017) and that the improvement of mesophyll behavior may be an important criterion to enhance the flood resistance of greengram cultivars (Araki et al., 2014). According to the previous findings, our data showed that both *g*_s_ and *g*_m_ regulate simultaneously *A* as a function of the total conductance to CO_2_ (Sorrentino et al., 2016; Santaniello et al., 2017; Scartazza et al., 2017). Herrera et al. (2008) reported that leaves of *Campsiandra laurifolia* Benth. developed under full flood exhibited a thicker mesophyll compared to leaves developed after falling water, possibly leading to a reduced *g*_m_ due to a longer diffusion path for CO_2_. Ren et al. (2016) showed that waterlogging negatively affects leaf mesophyll ultrastructure and photosynthetic characteristics in summer maize, suggesting an impact of waterlogging on membrane integrity leading to chloroplast, mitochondria and membrane deterioration that increased with increasing waterlogging duration. These alterations could affect CO_2_ diffusion through cell and chloroplastic membranes, thus reducing the mesophyll conductance (Flexas et al., 2008).

The ability to recover the photosynthetic performance after submergence or waterlogging treatment is a valuable trait in order to select the most tolerant species and genotypes to reduced O_2_ availability. In the present work, only 10 days of waterlogging treatment showed sustained reduced photosynthetic activity after 72 h of recovery, while submergence induced a reduction in photosynthesis after just seven DOT. It is worth noting that notwithstanding the full recovery of photosynthetic parameters, plant biomass remained reduced compared to the control depending on treatment and duration. Accordingly, in a previous work of Smethurst et al. (2005) on *Medicago sativa* L., PSII photochemistry, which was impaired due to waterlogging, recovered almost completely after draining alongside the concentrations of several nutrients, although growth remained suppressed. These authors attributed the reduced growth to both the smaller CO_2_ assimilation during waterlogging, due to nutrient deficiency and associated inhibition of PSII photochemistry, and the plant’s need to redirect available nutrient and assimilate pools to repair the damage to the photosynthetic apparatus and roots. In addition, the reduction in leaf area in plants subjected to waterlogging could also substantially contribute to a decrease of the photosynthetic area and hence plant biomass (Malik et al., 2001).

MFA enabled the set of observations based on chlorophyll *a* fluorescence and leaf gas exchange data to be analyzed within the same framework, thus giving an integrated picture of the observations and the relationships between the variables recorded at the beginning and end of the time-course recovery experiment. The analysis led to the gradual separation of the entries as affected by incremental O_2_ deficiency conditions with respect to the performance obtained. Moreover, the use of the multicanonical analysis highlighted the presence of specific thresholds (7 and 10 DOT, respectively, for submergence and waterlogging), which implied a treatment tolerance to the discrimination of the treatments.

## Conclusion

In the present study, *A. donax* confirmed its ability and a distinct response strategy that allowed the species to cope with harsh stress conditions. Plants subjected to waterlogging showed similar growth capacity to those under normoxia, while plants fully submerged showed a dramatic reduction of this trait. Conditions of waterlogging and submergence revealed a slight growth plasticity of the species in response to prolonged stress conditions, followed by fast plant recovery upon reoxygenation. Moreover, the rapid restoration of physiological functions during the recovery period after O_2_ deprivation testifies to the environmental plasticity of this species, although prolonged scarcity of O_2_ proved detrimental to giant reed by hampering growth and photosynthetic CO_2_ uptake. Those responses are today biologically and ecologically relevant for a species that has been promoted to marginal, degraded lands, and should be selected based on their performance under less than favorable conditions.

## Author Contributions

AP, AS, and LG conceived and designed the experiments. AP, AS, THR, and TM performed the experiments. AP, AS, and LG analyzed the data. AP, AS, LG, and THR wrote the paper.

### Conflict of Interest Statement

The authors declare that the research was conducted in the absence of any commercial or financial relationships that could be construed as a potential conflict of interest.
